# Incidence of post-operative infections and antibiotic resistance trends: A clinical study

**DOI:** 10.6026/973206300223594

**Published:** 2026-06-30

**Authors:** Vidya Kiran Bammidi, Jagannath Mishra, Dilip Katakam, Shamurailatpam Priyadarshini, Deepthi Nirmal Gavarraju, Neha Akhilesh Kumar Singh, Heena Dixit Tiwari

**Affiliations:** 1 Department of General Surgery, Gayatri Vidya Parishad Medical College, Visakhapatnam, Andhra Pradesh, India; 2 Department of Anaesthesiology, Government Medical College and Hospital, Sundargarh, Odisha, India; 3 Department of Conservative Dentistry and Endodontics, ESIC Dental College and Hospital, Kalaburagi, Karnataka, India; 4 Departments of Conservative Dentistry and Endodontics, Dental College, Regional Institute of Medical Sciences (RIMS), Imphal, Manipur, India; 5 Department of Pedodontics and Preventive Dentistry, Sibar Institute of Dental Sciences, Guntur Andhra Pradesh, India; 6 Department of Pathology, Central Government Health Services Wellness Centre-1, Bhubaneswar, Odisha, India; 7 Commissionerate of Health and Family Welfare, Government of Telangana, Hyderabad, India

**Keywords:** Surgical site infection (SSI), post-operative infection, antimicrobial resistance (AMR), antibiotic stewardship, hospital infection surveillance

## Abstract

Gram-negative organisms, particularly Escherichia coli and Klebsiella species, are predominant pathogens. Therefore, it is of interest to evaluate the microbiological culture reports, antimicrobial susceptibility testing and clinical records of surgical patients who developed post-operative infections. In our study 200 surgical patients were included, among 32 developed post-operative infections, so giving an overall infection incidence of 16%. High resistance rates were observed against commonly prescribed antibiotics including amoxicillin-clavulanate and ceftriaxone, whereas lower resistance was noted for carbapenems. Thus, data shows the increasing burden of antimicrobial resistance in post-operative infections and the importance of continuous microbiological surveillance for effective infection control.

## Background:

Post-operative infections, particularly surgical site infections (SSIs), remain among the most common complications following surgical procedures worldwide. These infections significantly contribute to prolonged hospital stays, increased treatment costs and higher morbidity and mortality among surgical patients. Despite advances in surgical techniques, aseptic precautions and perioperative antibiotic prophylaxis, the burden of post-operative infections continues to challenge healthcare systems globally [1]. Studies suggest that SSIs account for nearly 20% of all healthcare associated infections and represent one of the leading causes of preventable post-surgical complications [2]. The epidemiology of post-operative infections varies across regions depending on surgical practices, infection control policies and microbial ecology within healthcare settings. In low-and middle-income countries, the incidence of surgical site infections has been reported to range between 5% and 30%, significantly higher than that reported in high-income countries [3]. These infections are commonly caused by pathogens such as *Staphylococcus aureus, Escherichia coli, Klebsiella species *and Pseudomonas aeruginosa, many of which demonstrate increasing antimicrobial resistance [4]. Antimicrobial resistance (AMR) has emerged as a critical global health threat and is increasingly associated with hospital-acquired infections including SSIs. The widespread and often inappropriate use of antibiotics has accelerated the development of resistant bacterial strains, complicating treatment options and clinical outcomes [5]. Multidrug-resistant organisms such as methicillin-resistant *Staphylococcus aureus*(MRSA) and extended-spectrum beta-lactamase (ESBL) producing gram-negative bacteria have been frequently identified in post-operative infections [6]. Recent research has emphasized the importance of continuous surveillance of infection rates and antimicrobial susceptibility patterns to guide clinical decision-making and improve infection control strategies [7]. Monitoring trends in antibiotic resistance is essential for optimizing empiric antibiotic therapy and reducing the risk of treatment failure. In addition, antimicrobial stewardship programs have been increasingly promoted to ensure rational antibiotic use and limit the spread of resistant organisms within hospital environments [8]. Several studies have highlighted the importance of local epidemiological data in understanding the patterns of post-operative infections and resistance trends. Such data enable healthcare institutions to implement targeted infection prevention strategies and refine antibiotic prophylaxis protocols [9]. Moreover, updated clinical evidence indicates that regular surveillance of microbial pathogens and their susceptibility profiles can significantly improve patient outcomes and reduce healthcare-associated infection rates [10]. Therefore, it is of interest to evaluate the incidence of post-operative infections and analyze antibiotic resistance patterns among patients undergoing surgical procedures in a tertiary care hospital.

## Materials and Methods:

This retrospective observational study was conducted in the Department of Surgery of a tertiary care teaching hospital over a period of one year. The study included patients who underwent elective or emergency surgical procedures and subsequently developed clinical features suggestive of post-operative infection. Ethical approval was obtained from the institutional ethics committee prior to data collection. Patients aged above 18 years who developed signs of surgical site infection within 30 days following surgery were included in the study. Patients with pre-existing infections or incomplete medical records were excluded from analysis. Clinical data were obtained from hospital records including demographic characteristics, type of surgery performed, duration of hospital stay and post-operative complications. Specimens from suspected surgical site infections were collected under aseptic conditions and sent to the microbiology laboratory for culture and sensitivity testing. Samples included wound swabs, pus aspirates and tissue specimens. Microbial identification was performed using standard microbiological techniques including Gram staining, culture on selective media and biochemical testing. Antimicrobial susceptibility testing was conducted using the Kirby-Bauer disk diffusion method in accordance with Clinical and Laboratory Standards Institute (CLSI) guidelines. The antibiotics tested included commonly used agents such as ceftriaxone, ciprofloxacin, amoxicillin-clavulanate, piperacillin- tazobactam, meropenem and vancomycin. Resistance patterns were interpreted based on standard susceptibility criteria. Data were entered and analyzed using SPSS software version 25. Descriptive statistics were used to summarize demographic characteristics, infection rates and bacterial isolates. Categorical variables were expressed as frequencies and percentages. The incidence of post-operative infection was calculated as the proportion of infected cases among total surgical procedures performed during the study period.

## Results:

A total of 200 surgical patients were included in the study, among whom 32 developed post-operative infections, giving an overall infection incidence of 16%. The majority of infections occurred in patients undergoing abdominal surgeries, followed by orthopedic and general surgical procedures. Culture results revealed a predominance of gram-negative bacteria, with *Escherichia coli and Klebsiella* species being the most common pathogens isolated. Table 1 presents the demographic distribution of patients who developed post-operative infections. The majority of infected patients were within the age group of 41-60 years, accounting for 43.7% of cases. Male patients constituted a slightly higher proportion of infected individuals compared with females. Elderly patients above 60 years also demonstrated a considerable infection rate. The findings suggest that middle-aged and older individuals may be at increased risk of post-operative infections, possibly due to comorbidities and decreased immune response. Table 2 shows the distribution of surgical procedures among patients who developed infections. Abdominal surgeries accounted for the highest proportion of post-operative infections, representing more than half of the cases. Orthopedic surgeries were the second most common category associated with infections, followed by general surgical procedures. The higher infection rate observed in abdominal surgeries may be related to the complexity of procedures, contamination risk and longer operative duration compared with other surgical interventions. Table 3 illustrates the distribution of bacterial isolates obtained from infected surgical wounds. Gram-negative organisms predominated among isolates; with *Escherichia coli* representing the most frequently identified pathogen. *Klebsiella* species and *Staphylococcus aureus* were also commonly detected. The presence of both gram-positive and gram-negative pathogens highlights the polymicrobial nature of surgical site infections and emphasizes the importance of microbiological culture for appropriate antimicrobial therapy. Table 4 summarizes antibiotic resistance patterns among the isolated pathogens. High resistance rates were observed against commonly used antibiotics such as amoxicillin-clavulanate and ceftriaxone. In contrast, lower resistance rates were noted for carbapenems and vancomycin. The findings indicate a growing trend of antimicrobial resistance among pathogens responsible for post-operative infections, emphasizing the need for judicious antibiotic use and regular surveillance of susceptibility patterns in healthcare institutions.

## Discussion: 

Post-operative infections remain one of the most significant complications following surgical procedures and represent a major contributor to healthcare-associated infections worldwide [11, 12, 13]. In the present study, the overall incidence of post-operative infections was found to be 16%, which falls within the range reported in previous literature. Patangia *et al.* reported that surgical site infections account for a substantial proportion of hospital-acquired infections, particularly in developing healthcare systems where infection control measures may be inconsistently implemented [2]. The distribution of infections across age groups in this study demonstrated that patients between 41 and 60 years were most frequently affected. This finding may be attributed to the higher prevalence of comorbidities such as diabetes, hypertension and obesity in middle-aged populations, which can impair immune response and delay wound healing. Lakoh *et al.* similarly observed that advanced age and underlying medical conditions were significant predictors of surgical site infections in tertiary hospitals [3, 14, 15, 16]. In the present study, abdominal surgeries were associated with the highest proportion of infections. These procedures often involve contamination risk due to exposure to gastrointestinal flora, which increases the likelihood of infection. Rezaei *et al.* reported comparable findings in their investigation of post-operative infections following abdominal procedures, noting that surgical complexity and operative duration significantly influence infection risk [5]. The authors emphasized that prolonged surgical time and intraoperative contamination contribute to the development of post-operative infections. Microbiological analysis in this study revealed a predominance of gram-negative organisms, particularly *Escherichia coli* and *Klebsiella* species. This observation aligns with recent studies that have identified gram-negative bacteria as major causative agents of surgical site infections. Worku *et al.* reported that *E. coli* and *Klebsiella* were among the most frequently isolated organisms in post-operative wound infections, reflecting their widespread presence in hospital environments and their ability to develop resistance to multiple antibiotics [7]. Another important finding of the present study is the significant level of resistance observed among bacterial isolates to commonly used antibiotics such as amoxicillin-clavulanate and ceftriaxone. The increasing prevalence of antimicrobial resistance poses a serious challenge in the management of post-operative infections. The global burden of antimicrobial resistance has been extensively documented by the antimicrobial resistance collaborators, who highlighted that resistant bacterial infection; contribute substantially to global morbidity and mortality [10, 17]. Recent research has emphasized the role of antibiotic stewardship programs in addressing this challenge. Righi *et al.* highlighted that appropriate perioperative antibiotic prophylaxis and strict adherence to infection prevention protocols can significantly reduce the incidence of surgical site infections [9]. The present study also aligns with global observations emphasizing the need for updated antibiotic stewardship programs and the judicious use of prophylactic antibiotics [18]. Similarly, Long *et al.* reported that optimized antibiotic selection and timely administration play a crucial role in minimizing post-operative infection risk and preventing the emergence of resistant organisms [15]. The findings of this study also highlight the importance of continuous surveillance of antimicrobial resistance patterns. Monitoring resistance trends allows clinicians to select effective empiric antibiotics while minimizing unnecessary antimicrobial exposure. Studies have demonstrated that hospital-based infection surveillance programs can significantly improve patient outcomes by enabling early identification of emerging resistant pathogens [17, 19, 20]. Overall, the results of the present study underscore the need for strengthened infection prevention measures, including strict adherence to aseptic surgical techniques, proper antibiotic prophylaxis and implementation of antimicrobial stewardship programs. Regular monitoring of microbial profiles and resistance patterns is essential for guiding clinical management and improving surgical outcomes.

## Conclusion: 

Post-operative infections remain a significant challenge in surgical practice and contribute to increased morbidity and healthcare burden. The study demonstrated a considerable incidence of infections with a predominance of gram-negative pathogens and notable antimicrobial resistance patterns. Thus, continuous infection surveillance and rational antibiotic stewardship are essential to reduce post- operative infections and limit the spread of resistant organisms.

## Advancement to knowledge: 

This study provides updated local epidemiological evidence regarding the incidence, microbial profile and antimicrobial resistance patterns of post-operative infections in a tertiary care setting, thereby supporting evidence-based antibiotic stewardship, optimization of perioperative antibiotic protocols and strengthening of hospital infection surveillance systems.

## Figures and Tables

**Table 1:** Demographic distribution of patients with Post-operative Infection

**Age Group**	**Frequency**	**Percentage**
18-40	9	28.1
41-60	14	43.7
>60	9	28.1
Total	32	100

**Table 2:** Type of surgical procedure

**Surgery Type**	**Frequency**	**Percentage**
Abdominal Surgery	17	53.1
Orthopedic Surgery	9	28.1
General Surgery	6	18.8
Total	32	100

**Table 3:** Bacterial Isolates

**Organism**	**Frequency**	**Percentage**
*E. coli*	10	31.2
*Klebsiella spp.*	8	25
*Staphylococcus aureus*	7	21.9
*Pseudomonas aeruginosa*	5	15.6
Others	2	6.3

**Table 4:** Antibiotic resistance pattern

**Antibiotic**	**Resistant(%)**
Amoxicillin-Clavulanate	62.5
Ceftriaxone	56.2
Ciprofloxacin	40.6
Piperacillin-Tazobactam	28.1
Meropenem	12.5
